# Could pelvic parameters determine optimal postoperative thoracic kyphosis in Lenke type 1 AIS patients?

**DOI:** 10.1186/s12891-018-1992-z

**Published:** 2018-03-07

**Authors:** Shunan Liu, Yuancheng Zhang, Hongda Bao, Peng Yan, Zezhang Zhu, Zhen Liu, Bangping Qian, Yong Qiu

**Affiliations:** Spine Surgery, Nanjing Drum Town Hospital, Nanjing University Medical School, Nanjing, 210008 China

**Keywords:** Adolescent idiopathic scoliosis, Lenke type 1, Thoracic kyphosis, Pelvic parameters

## Abstract

**Background:**

A proper restoration of sagittal alignment is essential in AIS patients, but few studies provided a formula to predict an optimal surgical thoracic kyphosis (TK) gain in adolescent idiopathic scoliosis (AIS) patients. A formula was recently proposed (LL = (PI+TK)/2 + 10) to predict the optimal lumbar lordosis (LL) in adult spinal deformity patients, which has not been validated in adolescents. The aim of this study is to establish a formula with TK and pelvic parameters in normal adolescents and predict an optimal TK with this formula pre- and post-operatively in Lenke 1 AIS patients.

**Methods:**

A total of 60 asymptomatic adolescents were used to validate the proposed formula. The subject was considered to match with the formula, if the difference between the virtual TK and the theoretical TK was less than 10°. Then regression analysis was performed to establish a new formula to predict TK in adolescents. The predictive efficiency of the new formula was also validated in 40 Lenke 1 AIS patients.

**Results:**

Of the 60 asymptomatic adolescents, only 26 (43.33%) asymptomatic adolescents matched with the adjusted formula: TK = 2 × (LL-10)-PI. The paired *t* test revealed a significantly different theoretical TK (tTK) compared to the virtual TK (41.23 ± 18.29° vs. 24.80 ± 8.75°, *P* < 0.001). Multiple linear regression showed that TK had a relationship with LL, SS and age (R^2^ = 0.331): TK = − 0.785 × LL-0.843 × SS + 0.858 × age + 3.754. There were 27 (67.50%), 32 (80.00%) and 35 (87.50%) Lenke 1 AIS patients matched this formula preoperatively, postoperatively and at the last follow-up.

**Conclusion:**

Our results revealed that the predictive formula for sagittal alignment for adults was not applicable in adolescents. This study established a new predictive formula for TK based on asymptomatic adolescents. In Lenke 1 AIS patients, post-op TK in 87.5% of patients matched the predictive value, indicating that the new formula can be considered as a reference when making a surgery strategy.

## Background

After coronal realignment of adolescent idiopathic scoliosis (AIS) patients had been well studied, attention was then attracted to the sagittal alignment which may affect the health-related quality of life (HRQOL) in AIS patients after skeletal maturity [1, 2]. The relationship between sagittal parameters, composed of thoracic kyphosis (TK), lumbar lordosis (LL), pelvic incidence (PI), pelvic tilt (PT), and sacral slope (SS), has been revealed but detailed interactions between each other were still warranted. Qiu et al. [3] suggested that TK was strongly correlated with both LL and the upper arc of the LL in adolescent idiopathic thoracic scoliosis (T-AIS) patients and general population. Clément et al. [4] indicated that the proximal part of the lumbar lordosis depends on the thoracic hypokyphosis and the distal part depends on the pelvic incidence in thoracic AIS patients. Hypokyphosis in Lenke 1 AIS patients has been correlated to decreased HRQOL not only during adolescence but also in adulthood. Bernstein et al. [5] also revealed that the failure of kyphosis restoration after scoliosis surgery predisposed to lumbar disc degeneration during the follow-up. Thus, a proper restoration of TK is essential for Lenke 1 AIS patients.

The restoration of sagittal alignment was also emphasized in adult spinal deformity (ASD) patients which was demonstrated to benefit post-op HRQOL with less complication. A panel of experts on sagittal spinal deformity analyzed the sagittal parameters in ASD patients and proposed a formula: LL = (PI+TK)/2 + 10 [6], aiming to predict the optimal LL based on both PI and TK. For accurate restoration of the sagittal alignment, it is necessary to make a proper surgical plan which sets up a target of postoperative TK before the surgery. Since LL and TK are related with each other, this formula was adjusted to another form: TK = 2 × (LL-10)-PI. Because the formula was established based on adult patents, it need to be validated in normal adolescents before used in AIS patients. If it is not proper in adolescents, a new formula which was fit for adolescent was required. Thus, the aim of this study is to establish a formula with thoracic kyphosis (TK) and pelvic parameters in normal adolescents and predict an optimal TK with this formula pre- and post-operatively in Lenke 1 adolescent idiopathic scoliosis (AIS) patients.

## Methods

This is a retrospective radiographic study. Inclusion criteria consisted of Lenke 1 AIS patients, female, selective thoracic posterior fusion, at least 1-year follow-up, complete standing whole spine radiographs. Selective thoracic fusion is defined as LIV at L1 and above. The decision of selective fusion is based on the Lenke rule, including Lenke 1B cases with high Risser grade and Lenke 1A cases. The selection of LIV is mainly based on the experience of the surgeon, some cases followed the Lenke Touching Vertebra rule and some followed the Cobb to Cobb rule. The exclusion criteria include: history of any spinal surgery; any comorbidity which probably affected the spino-pelvic alignment such as congenital dysplasia of hip; incompleteness of patients’ information or lack of some measurements. Finally, 40 female patients with Lenke 1 AIS were included in this study from March 2014 to November 2015 at least 1 year follow-up (17.28 ± 7.33 months; range, 12–48 months). The average age of the patients was 14.33 years (range, 11–18 yr). Another 60 asymptomatic adolescent girls were also included. These girls presented to the clinic because of complaints about minor back asymmetry but were found to have normal spinal alignment from the standing whole spine radiographs. The average age of these volunteers was 12.63 years (range, 10–18 yr). This study was approved by the Clinical Research Ethics Committee of the hospital. Informed consent was obtained from every patient included in the study. If the patients were less than 18 years, the consents would be acquired from their parents.

**Fig. 1:**
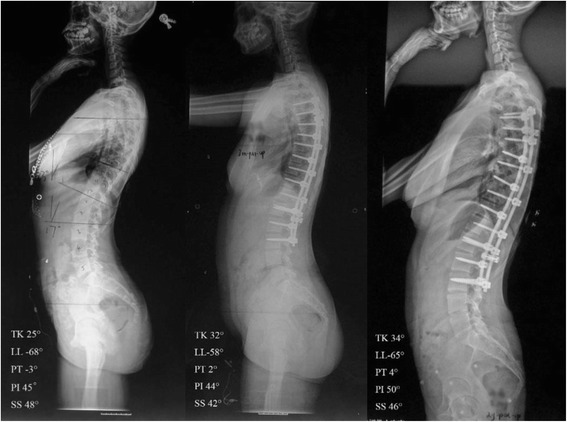
This 14-year-old girl presented with adolescent idiopathic scoliosis. The main thoracic Cobb angle was 41°. The pre-, post-operative and 2-year follow-up sagittal parameters were shown in the radiograph. The oTK calculated from the formula were 28.68°, 25.89° and 28.01° respectively

**Fig. 2:**
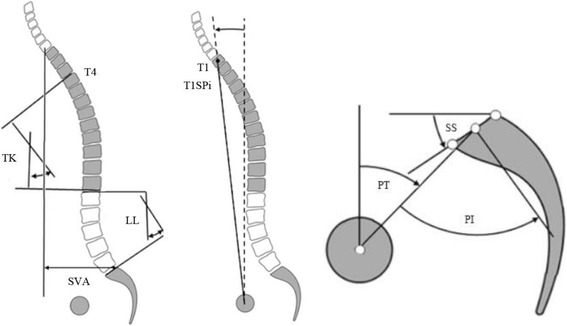
Sagittal Spinal radiologic parameters. TK is the angle between the cranial endplate of T4 and the caudal endplate of T12. LL is the angle between the upper endplate of L1 and the sacral endplate. SVA is the horizontal offset from C7 plumb line (C7PL) to the postero-superior cornor of S1. T1SPI is the angle between vertical and a line from the center of the femoral head axis to the center of the T1 vertebral body. PI is the angle between the line perpendicular to the sacral plate at its midpoint and the line connecting this point to the axis of the femoral heads. PT is the angle between the line connecting the midpoint of the sacral plate to the femoral heads axis and the vertical

Standing whole spine radiographs were performed preoperatively, postoperatively, at the last follow-up in AIS patients (Fig. 1). and in asymptomatic teenagers. All participants underwent the imaging examination in the fist-on-clavicle position [7]. The radiographs taken with wrong positon or hard to distinguish the vertebra end plate were excluded from this study. Sagittal parameters were measured on the standing lateral radiograph with image analysis software (Surgimap, Nemaris Inc., New York, NY, USA) (Fig. 2). The measured radiographic parameters were as follow:

PI is the angle between the line perpendicular to the sacral plate at its midpoint and the line connecting this point to the axis of the femoral heads. PT is the angle between the line connecting the midpoint of the sacral plate to the femoral heads axis and the vertical. SVA is the horizontal offset from C7 plumb line (C7PL) to the postero-superior cornor of S1. TK is the angle between the cranial endplate of T4 and the caudal endplate of T12. LL is the angle between the upper endplate of L1 and the sacral endplate. T1SPI is the angle between vertical and a line from the center of the femoral head axis to the center of the T1 vertebral body.

The International Spine Study Group (ISSG) formula [6] was transformed into another form: TK = 2 × (LL-10)-PI and validated in 60 asymptomatic adolescent volunteers. The theoretical TK (tTK) was calculated by LL and PI. If the difference between the tTK and virtual TK was less than 10°, this patient could be considered to match with the formula. If most of the patients didn’t confirm to the formula, a new formula would be established with multiple linear regression. Either the adjusted formula or the new formula would be used to predicted the optimal TK (oTK) in Lenke 1 AIS patients pre- and post-operatively after validation in asymptomatic adolescents.

### Statistical analysis

Data were presented as mean ± SD. Comparison of means used the independent-samples *t* test between AIS patients and volunteers. Paired *t* test was performed to evaluate the differences between the virtual TK and the TK calculated by formulas. Multiple linear regression analysis with a stepwise condition was s performed in asymptomatic group and established the formula predicting the optimal TK in AIS group. The independent variable of the multiple linear regression included age, LL, PT, PI and SS. Statistical tests were performed using SPSS (Inc. Version 22.0, Armonk, NY, IBM Corp). *P* < 0.05 was considered statistically significant.

## Results

A total of 60 asymptomatic adolescents and 40 Lenke 1 AIS patients were included in this study. The mean age of 12.63 years in the asymptomatic group is significantly smaller compared to that of 14.33 years in the AIS group (*P* < 0.001). The selection of the upper instrumented vertebra and the lower instrumented vertebra in the Lenke 1 AIS patients was shown in Table 1. The average preoperative Cobb angle of the AIS patients was 49.40 ± 9.88°, and the average postoperative Cobb angle was 13.1 ± 4.33°. The baseline radiographic parameters of the AIS patients and asymptomatic adolescents are listed in Table 2. LL, PI and SS were significantly lower in asymptomatic group than AIS group (*P* < 0.001). Besides, SVA was larger in asymptomatic population (− 28.76 mm VS. -13.41 mm, *P* < 0.004). No statistically significant difference was found regarding the remaining sagittal parameters between the two groups (all *p* > 0.05).

**Table 1:** The selection of UIV and LIV in the Lenke 1 AIS patients

	Level	n
UIV	T2	3
	T3	4
	T4	27
	T5	6
LIV	T12	2
	L1	38

*UIV* Upper instrumented vertebra, *LIV* Lower instrumented vertebra

**Table 2:** Sagittal parameters of the asymptomatic adolescents and the Lenke 1 AIS patients preoperatively

Parameters	Asymptomatic adolescents	Lenke 1 AIS patients	*P* Value
n	60	40	
TK (°)	24.80 ± 8.75	24.23 ± 13.56	0.813
LL (°)	− 50.82 ± 10.71	− 57.13 ± 12.12	0.007*
PI (°)	38.30 ± 10.87	47.30 ± 13.11	< 0.001*
PI-LL (°)	−12.52 ± 11.08	−9.85 ± 14.27	0.296
PT (°)	3.08 ± 9.39	6.43 ± 9.83	0.090
SS (°)	35.22 ± 8.09	40.88 ± 8.19	0.001*
T1SPI (°)	−5.20 ± 3.78	−4.98 ± 2.97	0.752
SVA(mm)	−28.76 ± 24.45	−13.41 ± 27.72	0.004*

*TK* Thoracic kyphosis, *LL* Lumbar lordosis, *PI* Pelvic incidence, *PI-LL* Lumbar-pelvic mismatch, *PT* Pelvic tilt, *SS* Sacrum slap, *T1SPI* T1 spino-pelvic inclination, *SVA* Sagittal vertical axis

*means the difference is statistically significant

As seen in Table 3, the mean measured TK was 21.90° postoperatively and 27.18° at the last follow-up, which increased significantly. After the surgery, the mean of LL dropped from 56.52° to 50.43°. The T1SPI of patients with Lenke 1 AIS at baseline was − 4.83°, which decreased to 2.33° postoperatively and increased up to 5.28° at follow-up (*P* < 0.001). Similarly, another sagittal balance parameter SVA was − 12.44 mm preoperatively, which turned to positive with a mean of 8.37 mm postoperatively and back to negative with a mean of − 14.77 mm (*P* = 0.001). However, PI, PI-LL, PT and SS were comparable during the follow-up.

**Table 3:** Sagittal parameters of the Lenke 1 AIS patients preoperatively, postoperatively and at last follow-up

	Preoperatively	Postoperatively	Follow-up	*P* value	Significance
TK (°)	23.33 ± 12.24	21.90 ± 7.77	27.18 ± 8.39	0.045	Post vs. FU
LL (°)	−56.52 ± 11.27	−50.43 ± 9.60	− 53.30 ± 11.04	0.041	Pre vs. Post
PI (°)	47.63 ± 12.84	46.90 ± 12.52	48.00 ± 13.52	0.928	–
PI-LL (°)	−8.9 ± 14.46	−3.53 ± 11.54	−5.30 ± 10.72	0.144	–
PT (°)	6.48 ± 9.79	6.93 ± 8.81	9.53 ± 8.80	0.277	–
SS (°)	41.15 ± 7.39	39.98 ± 8.02	38.48 ± 8.31	0.321	–
T1SPI (°)	−4.83 ± 3.00	−2.33 ± 2.71	−5.28 ± 2.99	< 0.001	Pre vs. Post and Post vs. FU
SVA(mm)	−12.77 ± 28.00	8.37 ± 31.04	−14.77 ± 29.02	0.001	Pre vs. Post and Post vs. FU

Only twenty six (43.33%) asymptomatic adolescents matched with the adjusted ISSG formula: TK = 2 × (LL-10)-PI. The paired *t* test showed that the theoretical TK (tTK) was significantly different from the virtual TK (41.23 ± 18.29° vs. 24.80 ± 8.75°, *P* < 0.001). The average difference between the virtual TK and tTK was 16.43 ± 18.04°. There were 10 (25.00%) AIS patients matching with the adjusted formula. Similarly, significant difference was found between the tTK and the virtual TK after the paired *t* test (46.95 ± 23.01° vs. 24.23 ± 13.56°, P < 0.001). The average difference between the two parameters was 22.73 ± 16.43°.

According to the asymptomatic subjects, multiple linear regression based on sagittal parameters of asymptomatic population revealed that TK had a relationship with LL, SS and age (R^2^ = 0.331): TK = − 0.785 × LL-0.843 × SS + 0.858 × age + 3.754. Then we did validation in AIS patients. There were 27 (67.50%), 32 (80.00%) and 35 (87.50%) Lenke 1 AIS patients matched this formula preoperatively, postoperatively and at the last follow-up. No significant difference was found between the virtual TK and the optimal TK (oTK) in any period of the AIS patients (*P* = 0.167, *P* = 0.265, *P* = 0.633, respectively) (Table 4). The average difference between the oTK and the virtual TK was 2.21 ± 9.89°, 1.32 ± 7.38°, − 0.54 ± 7.05° preoperatively, postoperatively and at the last follow-up.

**Table 4:** Paired comparison of TK and optimal TK in the Lenke 1 AIS patientst

		N	TK (°)	oTK (°)	oTK-TK (°)	*P* value
Lenke 1 AIS patients	Preoperatively	27/40 (67.50%)	23.33 ± 12.24	24.25 ± 7.86	2.21 ± 9.89	0.167
	Postoperatively	32/40 (80.00%)	21.90 ± 7.77	18.85 ± 5.69	1.32 ± 7.38	0.265
	Follow-up	35/40 (87.50%)	27.18 ± 8.39	25.54 ± 5.50	−0.54 ± 7.05	0.633

N the proportion of people who matched the formula in each group, TK thoracic kyphosis, oTK optimal thoracic kyphosis

## Discussion

Flatback syndrome often accounted as an iatrogenic complication of spine surgery including the correction of thoracic AIS may cause sagittal imbalance in the future [8]. Although few studies reported that the effect of hypokyphosis on short-term HRQoL in AIS patients, it was demonstrated that postoperative hypokyphosis might cause the decompensation of the cervical spine [9] or accelerated lumbar disc degeneration [5] during the long-term follow up. To avoid an abnormal postoperative TK, a formula was raised to predict an optimal TK with pelvic parameters which were expected to be independent from the corrective surgery.

The surgeons form ISSG [6] analyzed 137 adult spine deformity patients with optimal spinopelvic balance and established a formula: LL = (PI+TK)/2 + 10. We wondered if there were a similar correlation of LL, PI and TK in younger population so that we could predict an optimal TK in T-AIS patients after surgery. Therefore, the formula was adjusted into another form: TK = 2 × (LL-10)-PI and validated in the asymptomatic adolescents in this study. Unexpectedly, only 26 (43.33%) asymptomatic adolescents matched with the adjusted formula. The paired *t* test showed that the tTK was significantly different from the virtual TK (41.23 ± 18.29° vs. 24.80 ± 8.75°, *P* < 0.001). It might be because the ISSG formula was derived based on adults. Several studies suggested that TK, PI and PT were correlated with age [10–12], whereas the ISSG formula did not encounter the variables of age, potentially leading to the non-accurate prediction in younger population. In addition, different races could also lead to that result. PI, PT, and LL were found to be significantly greater in the African AIS patients when compared with the Caucasian AIS patients [13]. And a large amount of published data also showed that Asians have smaller values of LL and PI than Caucasians [12, 14–16]. The ethnical difference in terms of sagittal parameters may be the reason that the formula arisen from American population wasn’t accurate for Chinese population.

Thus, a new formula was programed to be established to predict ideal thoracic kyphosis. There were 60 asymptomatic adolescent girls recruited, in which the multiple linear regression was performed among the parameters and derived the formula: TK = − 0.785 × LL-0.843 × SS + 0.858 × age + 3.754. There were 67.50%, 80.00% and 87.50% patients matched the formula pre-, postoperatively and at the last follow-up, respectively. The average difference between the oTK and the virtual TK were small and not significant (2.21 ± 9.89°, 1.32 ± 7.38°, − 0.54 ± 7.05°). The postoperative virtual TK was a bit smaller than oTK indicating that the surgery might result in certain levels of hypokyphosis. The difference between the oTK and the virtual TK was continuous decreasing during the follow up possibly caused by the compensation of other segments and joints.

This is the first study which established a formula to predict an optimal TK with pelvic parameters in T-AIS patients. In ASD patients, the custom-made pre-contoured rod has been approved by the FDA and the clinical outcome was promising. Similarly, the predicted oTK could provide a reference for pre-contoured shape of the rod to obtain an optimal TK intra-operatively in AIS patients, achieving the tailor-made sagittal restoration. Stephen et al. [17] reported that T12 sagittal tilt combined with age could be used to predict thoracic kyphosis. But that study was performed in patients diagnosed with idiopathic scoliosis, degenerative scoliosis, kyphosis or some other diseases so that the TK predicted by T12 sagittal tilt might not be optimal. Kadoury et al. [18] published a nonlinear regression model based on the coronal thoracic curvature Cobb angle, the LL and the slope of the first lumbar vertebra to estimate the TK. The TK estimated by that model was also not optimal and couldn’t be referred in surgical planning. Since the pelvic parameters were not affected too much in thoracic selective fusion surgery, and pelvic parameters were similar between T-AIS patients and healthy girls [3], the formula derived from asymptomatic adolescent population was reasonable to predict an optimal TK in T-AIS patients. In addition, the formula included age as an independent variable due to the effect of age on sagittal parameters. It has been proved that TK, LL, PT and PI would gradually grow bigger with age during adolescence [19].

There were still several limitations in the current study. First, this study was a single-center study, which had a small sample size. Second, the age was not matched between the asymptomatic group and AIS group. In addition, because girls are more likely to suffer from AIS, to avoid the effects of gender on the formula, we only include female patients to establish the formula so that it may not be suitable for male AIS patients. Furthermore, the formula derived in this study need to be validated in other institutions and populations.

## Conclusions

Our results revealed that the predictive formula for sagittal alignment for adults was not applicable in adolescents. This study established a new predictive formula for TK based on asymptomatic adolescents. In Lenke 1 AIS patients, post-op TK in 87.5% of patients matched the predictive value, so it is applicable to use pelvic parameters and age to predict an optimal TK in asymptomatic population with the new formula. For Lenke 1 AIS patients, it is important to rebuild a proper TK after corrective surgery. The formula derived in this study provided a prediction of an optimal TK, which can be considered as a reference when making a surgery strategy.
